# Diversity of woodland strawberry inflorescences arises from heterochrony regulated by *TERMINAL FLOWER 1* and *FLOWERING LOCUS T*

**DOI:** 10.1093/plcell/koad086

**Published:** 2023-03-21

**Authors:** Sergei Lembinen, Mikolaj Cieslak, Teng Zhang, Kathryn Mackenzie, Paula Elomaa, Przemyslaw Prusinkiewicz, Timo Hytönen

**Affiliations:** Department of Agricultural Sciences, Viikki Plant Science Centre, University of Helsinki, PO Box 27, Helsinki FIN-00014, Finland; Department of Computer Science, University of Calgary, Calgary, AB, Canada T2N 1N4; Department of Agricultural Sciences, Viikki Plant Science Centre, University of Helsinki, PO Box 27, Helsinki FIN-00014, Finland; Department of Agricultural Sciences, Viikki Plant Science Centre, University of Helsinki, PO Box 27, Helsinki FIN-00014, Finland; Department of Agricultural Sciences, Viikki Plant Science Centre, University of Helsinki, PO Box 27, Helsinki FIN-00014, Finland; Department of Computer Science, University of Calgary, Calgary, AB, Canada T2N 1N4; Department of Agricultural Sciences, Viikki Plant Science Centre, University of Helsinki, PO Box 27, Helsinki FIN-00014, Finland

## Abstract

A vast variety of inflorescence architectures have evolved in angiosperms. Here, we analyze the diversity and development of the woodland strawberry (*Fragaria vesca*) inflorescence. Contrary to historical classifications, we show that it is a closed thyrse: a compound inflorescence with determinate primary monopodial axis and lateral sympodial branches, thus combining features of racemes and cymes. We demonstrate that this architecture is generated by 2 types of inflorescence meristems differing in their geometry. We further show that woodland strawberry homologs of *TERMINAL FLOWER 1* (*FvTFL1*) and *FLOWERING LOCUS T* (*FvFT1*) regulate the development of both the racemose and cymose components of the thyrse. Loss of functional FvTFL1 reduces the number of lateral branches of the main axis and iterations in the lateral branches but does not affect their cymose pattern. These changes can be enhanced or compensated by altering *FvFT1* expression. We complement our experimental findings with a computational model that captures inflorescence development using a small set of rules. The model highlights the distinct regulation of the fate of the primary and higher-order meristems, and explains the phenotypic diversity among inflorescences in terms of heterochrony resulting from the opposite action of *FvTFL1* and *FvFT1* within the thyrse framework. Our results represent a detailed analysis of thyrse architecture development at the meristematic and molecular levels.

## Teaser

*FLOWERING LOCUS T1* and *TERMINAL FLOWER 1* control the number of lateral branches and their branching iterations in the thyrse inflorescence of woodland strawberry.

**IN A NUTSHELL**

**Background:** Plants organize their flowers into clusters called inflorescences. The branching patterns and architecture of these clusters are crucial to a plant's reproductive success and agricultural yield. The extensive variability of inflorescence architectures in strawberries was documented a century ago. Since then, inconsistent characterizations of strawberry inflorescence architectures have accumulated. To clarify these inconsistencies, we studied the architecture and development of strawberry inflorescences at the macro and micro scales using diploid woodland strawberry (*Fragaria vesca*) as a model plant.

**Question:** We wanted to understand how the diversity of strawberry inflorescences is regulated at developmental and molecular levels.

**Findings:** Woodland strawberries produce compound inflorescences that consist of a primary monopodial axis bearing sympodial lateral branches. The primary axis and lateral branches are produced by geometrically distinct meristems. The resulting architecture, known as a thyrse, is not found in other previously studied model plants. The diversity of thyrse architecture originates from a variable number of lateral branches on the primary axis and a variable number of sympodial branching iterations. At the molecular level, both numbers are controlled by 2 homologous proteins, TERMINAL FLOWER 1 (FvTFL1) and FLOWERING LOCUS T1 (FvFT1), which regulate the timing of cessation of branching. The observed diversity of woodland strawberry inflorescences has been reproduced using a computational model that takes into account the functions of *FvTFL1* and *FvFT1*.

**Next steps:** Detailed molecular level mechanisms that drive the differences between the meristems and create thyrse architecture should be discovered to facilitate our understanding of the evolution of the morphological diversity in nature. This knowledge can be translated into the development of new breeding strategies to optimize the yield and quality of berries in cultivated strawberry.

## Introduction

**Figure 1.**
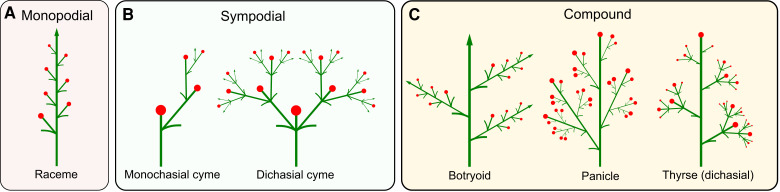
Classification of inflorescences based on their branching types. **A)** Monopodial branching of a simple raceme. **B)** Sympodial branching of monochasial and dichasial cymes. **C)** Compound branching of a botryoid, panicle, and thyrse.

The arrangement of individual flowers in time and space is critical for plants' reproductive success (Harder et al. 2004; Harder and Prusinkiewicz 2013), and ultimately for agricultural yield and fruit uniformity (Eshed and Lippman 2019). Many angiosperms organize their flowers into clusters called inflorescences. Based on their branching patterns, a vast variety of inflorescence architectures can be classified as monopodial or sympodial (Weberling 1989; Prusinkiewicz and Lindenmayer 1990). In monopodial inflorescences, or racemes (Fig. 1A), flowers form at the lateral positions on the indeterminate or determinate primary axis. In contrast, in cymose or sympodial inflorescences (Fig. 1B), flowers form at the terminal positions while new growth axes are established laterally. The classification of inflorescence architectures is complicated by the occurrence of compound inflorescences. For example, in compound racemes (e.g. botryoids) and panicles, the monopodial primary axis bears monopodial branches (Fig. 1C, left and center). In contrast, in thyrses, the primary indeterminate or determinate (Prenner et al. 2009) monopodial axis bears sympodial branches (Fig. 1C, right).

Woodland strawberry (*Fragaria vesca* L.) is a perennial rosette plant, a model for the octoploid cultivated strawberry (*Fragaria × ananassa*) and the Rosaceae family in general (Edger et al. 2018). Historically, the *Fragaria* inflorescence architecture was classified as pleiochasial cyme (Valleau 1918), dichasial cyme (Anderson and Guttridge 1982; Guttridge 1985), cyme (Menzel 2019), and corymb (Gleason and Cronquist 1991). This diversity of terms stems from the morphological variability of strawberry inflorescences, highlighted almost a century ago by Darrow (1929) and attributed to the environment, genetic differences between cultivars (Darrow 1929; Foster and Janick 1969; Anderson and Guttridge 1982) and sex of the flowers (Ashman and Hitchens 2000). Fundamentally, however, it points to the need for a resolution through a morphological analysis of inflorescence development at the meristem level.

Shoot apical meristems (SAMs) are small groups of multipotent cells located at the branch tips. Inflorescence meristems (IMs) are capable of initiating new IMs (i.e. branching axes) and, in determinate inflorescences, eventually transition to floral meristems (FMs), which produce flower organs. The molecular processes that regulate developmental decisions have been unraveled in several model plants with monopodial or sympodial inflorescences. In racemes, such as Arabidopsis (*Arabidopsis thaliana*), the IM state is maintained by the TERMINAL FLOWER 1 (TFL1) protein. Plants lacking functional *TFL1*, which is normally expressed at the center of the developing IM, produce determinate inflorescences with a few flowers (Alvarez et al. 1992; Schultz and Haughn 1993). On the IM flanks, the FM identity of the emerging primordia is defined by the expression of *LEAFY* (*LFY*) and *APETALA1* (*AP1*) genes, whose inactivation results in development of shoots instead of flowers (Weigel et al. 1992; Weigel and Meyerowitz 1993). The expression of *LFY* and *AP1* is activated by TFL1 homolog FLOWERING LOCUS T (FT) (Zhu et al. 2020), a mobile protein produced in leaves and transported to the SAM (Corbesier et al. 2007). The loss of functional FT was shown to promote IM indeterminacy in Arabidopsis and to increase the total number of flowers per inflorescence in the wild-type (WT) and *tfl1* plants (Lee et al. 2019).

As indicated by computational modeling, an interplay between *TFL1* and *LFY* can also result in other types of inflorescences, such as panicles and cymes (Prusinkiewicz et al. 2007). Nevertheless, genetic studies of pea (*Pisum sativum*) revealed an additional level of regulation. The pea inflorescences are heterothetic botryoids: the main axis supports second-order racemes, but does not end with a raceme (Benlloch et al. 2015). This architecture requires the identities of the primary and secondary IMs to be controlled separately. DETERMINATE (DET), a pea TFL1 homolog, functions similarly to its Arabidopsis counterpart maintaining the primary IM identity (Foucher et al. 2003). In contrast, the identity of the secondary IMs in pea is conferred by the expression of a MADS-box gene *VEGETATIVE1* (*VEG1*; Berbel et al. 2012), activated by GIGAS, a homolog of FT (Hecht et al. 2011). Pea homologs of AP1 and LFY then specify the FM identity of meristems produced on the flanks of the secondary IMs (Berbel et al. 2001).

Studies in plants with cymose inflorescences have revealed both similarities and differences in the genetic control of inflorescence development, compared to racemes. For example, orthologs of *LFY* in tomato (*Solanum lycopersicum*) and petunia (*Petunia hybrida*), both members of the *Solanaceae* family—*FALSIFLORA* (*FA*) and *ABERRANT LEAVES AND FLOWERS* (*ALF*), respectively—are expressed in the terminal meristems and promote the transition to the FM state (Molinero-Rosales et al. 1999; Souer et al. 2008). To fully establish FM identity, FA and ALF interact with cofactors, ANANTHA (AN) and DOUBLE TOP (DOT), respectively, both orthologous with the Arabidopsis UNUSUAL FLOWER ORGANS (UFO) (Chae et al. 2008; Lippman et al. 2008; Souer et al. 2008). Furthermore, *SINGLE FLOWER TRUSS* (*SFT*), a tomato ortholog of *FT*, reduces branching in both vegetative shoots and inflorescences, and promotes flowering (Quinet et al. 2006; Park et al. 2012). In contrast to its Arabidopsis counterpart, however, the tomato ortholog of *TFL1*, *SELF-PRUNING* (*SP*), controls the development of vegetative sympodial shoots, but does not affect the inflorescence architecture (Pnueli et al. 1998).

Here, we demonstrate that the strawberry inflorescence combines a monopodial primary axis with sympodial lateral branches, indicating that it is a thyrse. We show that the distinction between the monopodial and sympodial components of the inflorescence is associated with a different geometry of the respective meristems, and likely involves separate control of their fate. The complexity of both components is regulated by the strawberry homologs of *TFL1* and *FT,* which, respectively, increase and decrease the extent of branching. These findings lead to a computational model of thyrse development, which shows that the diversity of strawberry thyrses can be attributed to heterochrony, i.e. differences in the timing of the developmental transitions caused by the altered dosage of *FvFT1* and *FvTFL1*. Overall, our results present a detailed analysis of thyrse architecture development at the meristematic and molecular levels.

## Results

### Inflorescence of woodland strawberry is a determinate thyrse

**Figure 2.**
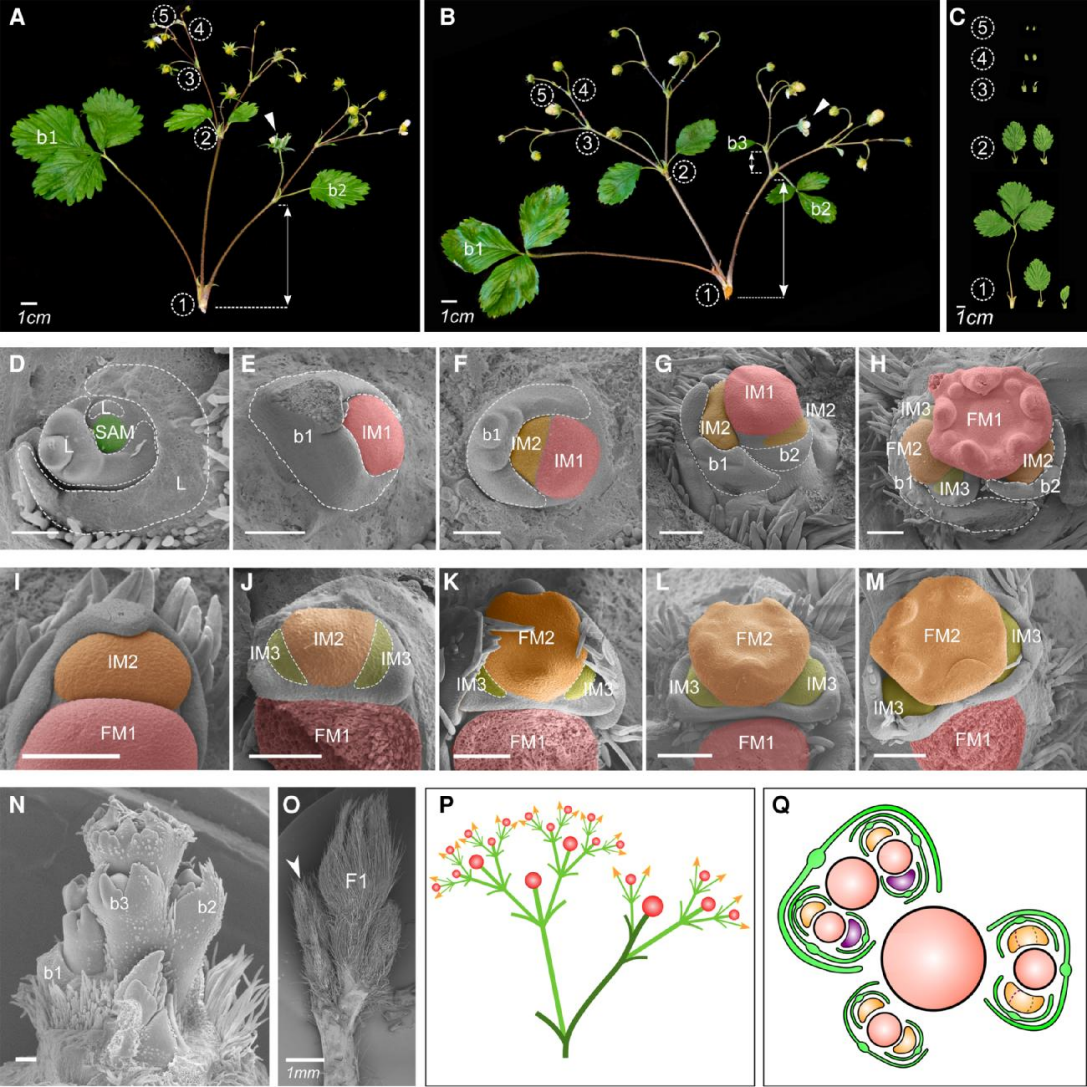
Architecture and development of thyrse inflorescence in woodland strawberry. Inflorescence architectures of WT woodland strawberry plants with 2 **A)** and 3 **B)** lateral branches on the primary axis. White arrowheads indicate the primary flowers; white lines with arrows indicate the distances between bracts on the primary branching axes; numbers in circles indicate branching iterations. **C)** Typical bract phenotypes at branching points of different orders. Development of the primary IM as observed by SEM. **D)** Round SAM with monopodially produced leaves. **E)** Early IM1 of increased size, compared to the SAM. **F)** Initiation of the first lateral IM2. **G)** Initiation of the second lateral IM2. **H)** IM1 transitioned into flower meristem (FM1). Development of the lateral meristems. **I)** Elongated, crescent-like shape of IM2. **J)** An almost simultaneous initiation of the lateral IM3s (dashed) at the ends of IM2. **K–M**) Transition of IM2 into FM2 and development of IM3s. **N)** Primary axis with 3 bracts, each subtending a lateral meristem. **O)** The first lateral branch (white arrowhead) displaces the primary flower (F1) and assumes a more dominant position. **P)** Schematic representation of thyrse inflorescence with leading lateral branch. The primary axis is highlighted in darker green. **Q**) Schematic representation of relative meristem positions during the development of woodland strawberry thyrse. Relatively more confined IMs are highlighted in purple. Scale bars in (D–N) equal 100 *µ*m. b, bract.

To understand the variability of strawberry inflorescences, we analyzed inflorescence development in diploid woodland strawberry, more conducive to genetic studies than the octoploid garden strawberry (*Fragaria × ananassa*). A woodland strawberry inflorescence has a monopodial primary axis terminated by a flower. This axis typically supports 2 (Fig. 2A) and occasionally 3 (Fig. 2B) secondary branches separated by long internodes. In many monopodial structures, the main axis is relatively straight and can be recognized easily. In woodland strawberry, however, secondary branches often assume a dominant position, continuing approximately in the direction of their supporting internodes, whereas the primary axis changes direction at each branching point. To identify the course of the primary axis we thus relied on the positions of bracts (b; Fig. 2) associated with the branches they subtend.

The architecture of lateral branches is different from that of the main axis. Each secondary axis supports a pair of third-order branches subtended by bracts, then terminates with a secondary flower. The third-order branches are approximately opposite each other: the internode between them is practically absent. This branching pattern repeats with each third-order branch producing a pair of fourth-order branches and a terminal flower, and typically continues up to fifth- or sixth-order branches. At high branching orders, only 1 (or none) lateral branch may emerge, although both bracts are present (Fig. 2, A and B; see also Jahn and Dana 1970). In spite of this departure from symmetry, the structures supported by the primary axis are best characterized as dichasial cymes (Fig. 1B, right). With the determinate primary monopodial axis supporting sympodial branches, inflorescence of woodland strawberry qualifies as a determinate thyrse (Fig. 1C, right).

Characterizing these inflorescences further, we observed that they are basitonic, i.e. the branches originating near the inflorescence base are more elaborate than those near the top (Lück et al. 1990). The first lateral branch growing from the axil of the leaf-like bract b1 is larger and bears more flowers than the branch that originates from the axil of b2 (Fig. 2, A and B). The third lateral branch, if present, is even smaller and produces fewer flowers than the second or the first lateral branch. The internode between b2 and b3 is also shorter than between b1 and b2 (Fig. 2, A and B). Moreover, the size of successive bracts is dramatically reduced, and the shape is simplified from a 3-lobed structure resembling a leaf to a bracteole-like structure with a single lobe (Fig. 2C). Within branches, the bracts are more similar in shape, but also decrease in size with each successive branching iteration.

### Two meristem types produce determinate thyrse of woodland strawberry

To investigate the ontogenesis of the strawberry inflorescence, we obtained a developmental sequence of IMs using scanning electron microscopy (SEM) imaging (Fig. 2, D to M). In woodland strawberry, the transition to flowering is associated with bulging and an almost 2-fold increase in the size of the primary IM (i.e. the meristem that generates the primary inflorescence axis: IM1), compared to the vegetative SAM (Fig. 2, D and E). IM1 has an approximately circular cross-section and produces lateral meristems (IM2) that are almost as large as IM1. The first bract (b1) is initiated during the SAM to IM1 transition (Fig. 2E), with a lateral meristem (IM2) forming in the bract axil (Fig. 2F). As the primary meristem (IM1) continues to grow, 1 or 2 additional lateral meristems (IM2) arise sequentially (Fig. 2, G, H, and N), leading to the monopodial architecture of the main axis. Due to their size, these bracts and meristems push the primary meristem (IM1) to the side (Fig. 2O), which changes the course of the main axis at each branching point (Fig. 2P) observed in the mature inflorescences (e.g. Fig. 2, A and B). The patterning of the main axis is terminated by the primary meristem acquiring flower identity and switching to the production of flower organs (FM1; Fig. 2H).

The second and higher-order IMs are morphologically distinct from the primary meristem. They extend along the perimeter of their parent meristems and have an elongated, crescent-like shape (Fig. 2, F and G). In consequence, the space needed to form the next-order meristems with associated bracts emerges concurrently near both poles (ends) of their parent meristem (Fig. 2, I and J). These primordia become meristems that may produce the next iteration of the same pattern, while the parent meristem continues to grow without changing direction and develops into a flower (Fig. 2, K to M). The dichasial structure of the woodland strawberry thyrse thus results.

Due to geometric constraints, 1 pole of each meristem of the third order (IM3) is inevitably closer to the inflorescence center than the other end, which leads to the uneven restriction of space available for the development of the fourth-order primordia (IM4; Fig. 2Q). A similar asymmetry occurs in higher order meristems, which may be the cause of, or contribute to, the commonly observed departure from symmetry at high branching orders, where only 1 lateral branch is present.

### *FvTFL1* is a major regulator of inflorescence architecture in woodland strawberry

In many plant species, inflorescence architecture and flowering time are tightly connected (Lifschitz et al. 2006; Liu et al. 2013; Lee et al. 2019). With this in mind, we looked for the genetic regulators that can affect architecture and development of the strawberry thyrse. In woodland strawberry, a mutation in *FvTFL1* accelerates flowering and reverses the photoperiodic requirement for flower induction from short- to long-day (Koskela et al. 2012). To understand whether *FvTFL1* also controls the woodland strawberry inflorescence development, we analyzed the inflorescence architecture and branching in 7 *fvtfl1* mutants and 9 WT genotypes collected across Europe (Supplemental Fig. S1, A to C). All *fvtfl1* cultivars used in this study were found to have a 2-bp deletion in the first exon of *FvTFL1* (Supplemental Fig. S2), putatively leading to a nonfunctional FvTFL1 protein.

**Figure 3.**
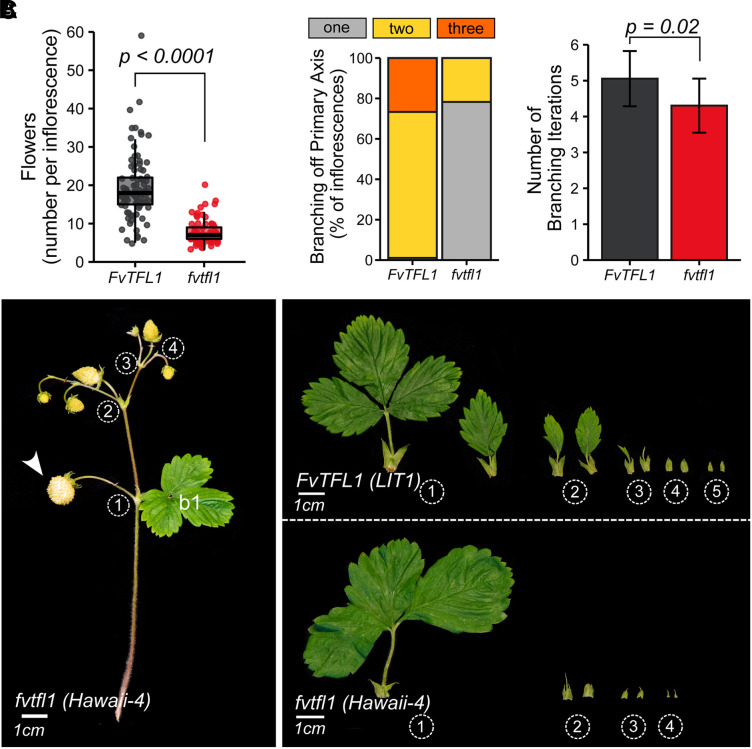
FvTFL1 controls inflorescence architecture in woodland strawberry. **A)** Number of flowers on the first inflorescence of 7 *fvtfl1* (red) and 9 *FvTFL1* (gray) genotypes. Boxplots and points show the distribution of raw data. Each point represents an individual inflorescence (*n* [*FvTFL1*] = 90 and *n* [*fvtfl1*] = 69). **B)** Percentage of observed inflorescences with 1, 2, or 3 branches on the primary branching axis. **C)** Average number of branching iterations along the longest branching path. Bars and error bars represent the mean ± Sd. Data in (A) and (C) were analyzed using a generalized linear mixed model (GLMM), with accessions and cultivars nested within *fvtfl1* and *FvTFL1* groups. *P* values for significant differences between *fvtfl1* and *FvTFL1* groups of plants are shown (pairwise *t-*test). **D)** Inflorescence phenotype of *fvtfl1* cultivar Hawaii-4; white arrowhead indicates the position of the primary flower. b, bract. **E)** Typical bract phenotypes along the longest branching path of *FvTFL1* and *fvtfl1* genotypes. Numbers in circles indicate branching iterations.

We found that the number of flowers per inflorescence was reduced more than 50% in the *fvtfl1* mutant plants. On average, WT plants produced 14.2 ± 1.2 flowers per inflorescence, while *fvtfl1* mutants produced only 6.5 ± 1.4 flowers per inflorescence (*P* < 0.0001; Fig. 3A). This reduction of flower numbers was associated with changes in the general architecture of inflorescences, particularly with dramatic changes in the number of lateral branches on the primary axis (Fig. 3, B to D). In plants with functional FvTFL1, we observed a high proportion of inflorescences with 2 (72%), and 3 (27%), lateral branches produced on the primary axis. Only 1% of the inflorescences had a single secondary branch. In contrast, the majority of inflorescences of *fvtfl1* mutant plants (78%) produced only 1 secondary branch (Fig. 3B) and the remaining 22% had 2 secondary branches. Such a dramatic change in the proportion of lateral branches supported by the primary axis suggests that in *fvtfl1* mutants IM1 transitions to FM1 before it is able to produce the second lateral branch on the primary axis.

In the lateral cymose branches, the loss of functional FvTFL1 did not affect the dichasial branching pattern. However, the total number of branching iterations in the *fvtfl1* mutants (4.3 ± 1.1) was reduced (5.1 ± 1.0; *P* = 0.02; Fig. 3C) and was associated with earlier reduction of bract size compared to the WT plants (Fig. 3E). The first bract (b1) on the primary axis was the largest and typically had 3 lobes in plants with functional and nonfunctional FvTFL1. Furthermore, in both genetic backgrounds, each successive lateral IM produced smaller bracts; however, at each corresponding branching point, WT plants had larger bracts than *fvtfl1* mutants.

### *FvFT1* regulates inflorescence architecture in *fvtfl1* background

**Figure 4.**
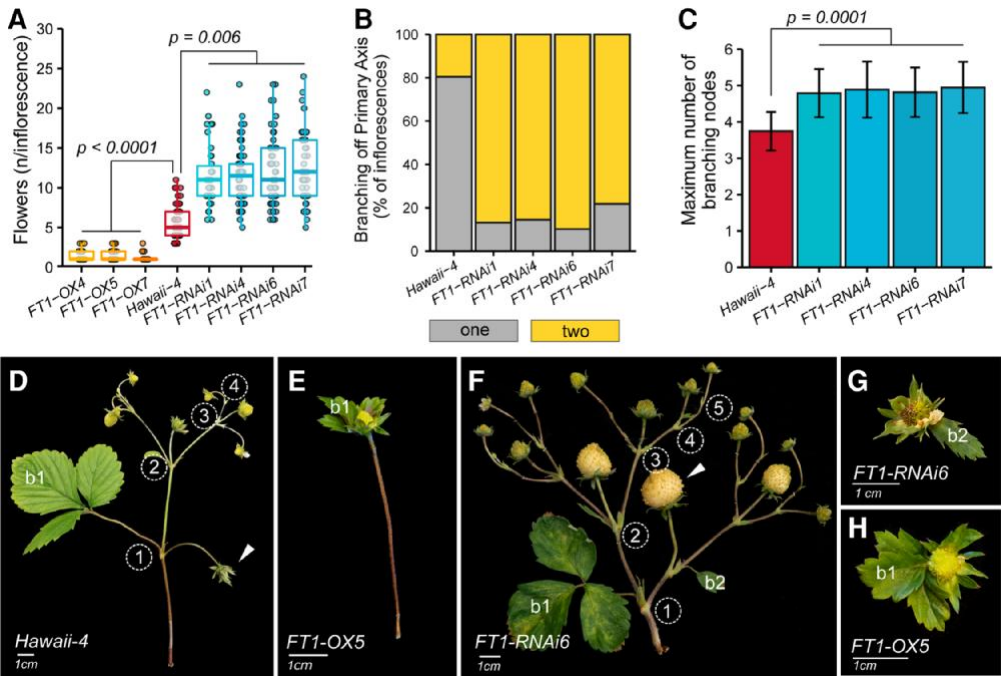
FvFT1 regulates inflorescence architecture in woodland strawberry. **A)** Number of flowers per inflorescence in *FvFT1* overexpression *(FT1-OX)*, *FvFT1* silenced (*FT1-RNAi*), and control *(Hawaii-4)* plants. Boxplots and points show the distribution of raw data. Each point represents an individual inflorescence. Up to 5 inflorescences per 8 to 13 plants in each line were examined. **B)** Percentage of inflorescences with 1 and 2 branches along the primary axis. **C)** Maximum number of branching iterations in *FvFT1-RNAi* lines and control *(Hawaii-4)* plants. Bars and error bars show the mean ± Sd of the raw data. Data in (A) and (B) were analyzed by fitting a generalized linear mixed model (GLMM); plants were grouped within construct type (OX, RNAi, or control). *P* values for significant differences between OX/RNAi and control are shown (Tukey's HSD). **D–F**) Inflorescence phenotypes of control *(Hawaii-4), FT1-OX5*, and *FT1-RNAi6* plants. The numbers in circles indicate branching iterations. White arrowheads indicate the primary flower. **G and H)** Intermediate phenotypes of *FvFT1-RNAi* and *FvFT1-OX* plants associating the second (b2) or first (b1) bract of the primary axis with the sepals of the primary flower. b, bract.

Next, we decided to investigate the role of *FvFT1* in the regulation of inflorescence architecture. Previously, FvFT1 was found to promote flowering under long-day conditions in the *fvtfl1* mutant background (Rantanen et al. 2014; Kurokura et al. 2017). We analyzed the inflorescence architecture and branching in *fvtfl1* plants (*Hawaii-4*) with silenced (RNAi) and overexpressed (*pro35S:FvFT1*) *FvFT1* (Supplemental Fig. S3). The cultivar *Hawaii-4* plants typically produced inflorescences with 5.7 ± 1.9 flowers, whereas plants overexpressing *FvFT1* (*FT1-OX*) produced inflorescences with only 1 to 3 flowers (*P* < 0.0001; Fig. 4, A, D, and E), in addition to dramatic reduction of the overall plant and leaf size due to quick inflorescence formation in all shoots (Supplemental Fig. S4).

In contrast, silencing of *FvFT1* caused an almost 2-fold increase in the number of flowers per inflorescence compared to Hawaii-4 (*FT1-RNAi*; 12 ± 0.24; *P* = 0.006; Fig. 4A). The main reason for the higher number of flowers per inflorescence was the formation of the second lateral branch along the primary axis, as in the genotypes with functional FvTFL1. We observed that about 80% of the inflorescences in *FvFT1-RNAi* plants initiated 2 lateral branches on the primary axis, compared to only 20% in *Hawaii-4* plants (Fig. 4B). Furthermore, *FvFT1-RNAi* plants typically produced more branching iterations than *Hawaii-4* (*P* = 0.0001 Fig. 4, C, D, and F). The branching of the primary axis in *fvtfl1* mutant plants (*Hawaii-4*) with silenced *FvFT1* expression was similar to WT plants with functional FvTFL1, except that we did not observe inflorescences with 3 lateral branches on the primary axis in *FvFT1-RNAi* lines (Fig. 4F). Moreover, we found that *FvFT1-RNAi* plants occasionally produced an intermediate phenotype, where the second bract (b2) on the primary axis was associated with the sepals of the primary flower (Fig. 4G). A similar phenotype was observed in the *FvFT1-OX* lines, however, in these lines the first bract (b1) became associated with the sepals of the primary flower (Fig. 4H).

In the plants with the functional FvTFL1, the overexpression of *FvFT1* did not cause as severe reduction in the overall plant size (Supplemental Fig. S5A) as in *FvFT1-OX fvtfl1* plants (Supplemental Fig. S4). The number of flowers per inflorescence of *FvFT1-OX* plants with functional FvTFL1 background was reduced compared to WT and *fvtfl1* mutant plants (Supplemental Fig. S3B), yet remained higher than in the *FvFT1-OX* plants with *fvtfl1* background. Altogether our data thus suggest the direct antagonistic functions of FvFT1 and FvTFL1 proteins in inflorescence development of woodland strawberry.

### *FvTFL1* and *FvFT1* control the rate of IM to FM transition in woodland strawberry

**Figure 5.**
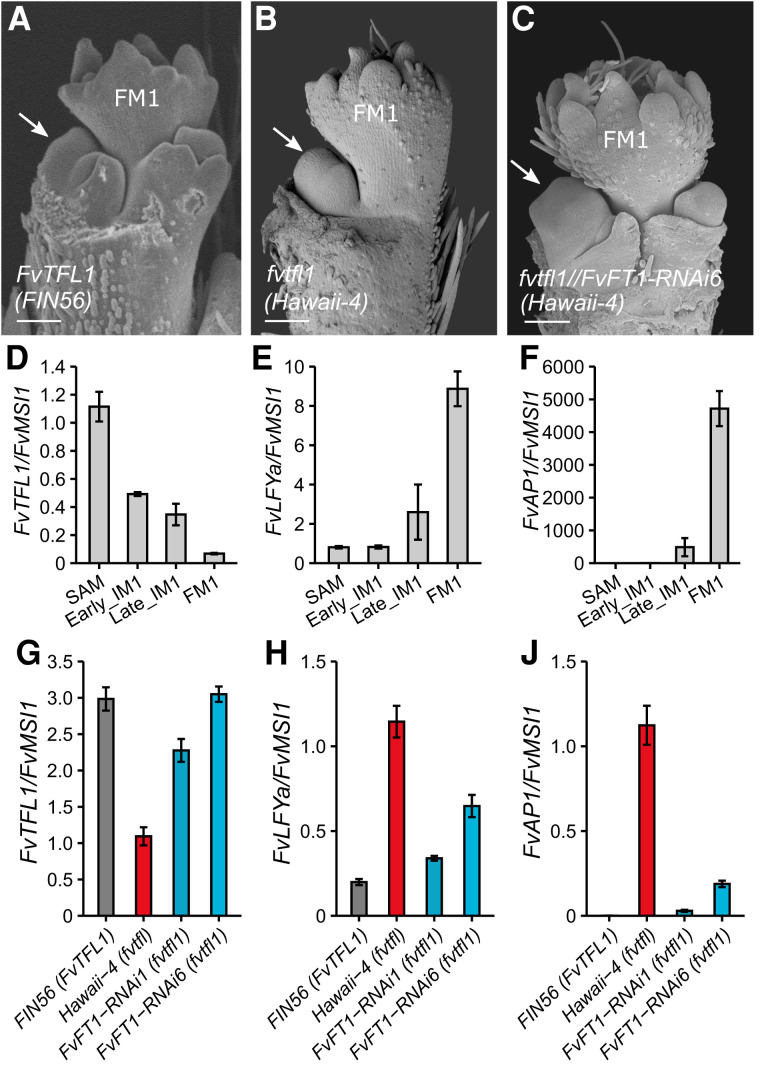
FvTFL1 and FvFT1 antagonistically shape the inflorescence of woodland strawberry. Primary axis development in *FvTFL1 WT* **A)**, *fvtfl1* mutant **B)**, and *FvFT1-RNAi* **C)** plants as observed by SEM. White arrows denote the first lateral IM2. Scale bars equal 100 *µ*m. The expression pattern of *FvTFL1* **D)**, *FvLFYa* **E)**, and *FvAP1* **F)** in different meristems of *FvTFL1 WT* (*FIN56*) during transition to flowering. The expression pattern of *FvTFL1* **G)**, *FvLFYa* **H)**, and *FvAP1* **J)** in the SAMs of FIN56 (WT), *Hawaii-4* (*fvtfl1*), and *FvFT1-RNAi* (*Hawaii-4*) plants. Bars and error bars show the mean ± Se (*n* = 3 to 5). Expression levels were normalized to those in the SAM (D–F) or *Hawaii-4* (G–J). *FvMSI1* was used as a calibrator gene.

To understand the mechanism of the second lateral branch formation on the primary axis we analyzed WT (*FIN56*), *fvtfl1* (*Hawaii-4*), and *FvFT1-RNAi fvtfl1* plants using SEM imaging (Fig. 5, A to C). We compared the WT and *fvtfl1* plants at a stage when the primary FMs showed a similar developmental phase. The first lateral meristem (IM2) of *FIN56* with functional FvTFL1 was at a later developmental stage (Fig. 5A) than the first (and single) lateral meristem (IM2) of *Hawaii-4* (Fig. 5B). In *FIN56*, the lateral IM2 already formed a pair of axillary meristems (IM3s) and sepal primordia, while in *Hawaii-4*, only the bracts could be clearly distinguished on the single lateral IM2. Similarly, the lateral meristem development was advanced when *FvFT1* was silenced in *fvtfl1* background (Fig. 5C). Overall, these findings suggest that the primary IM1 of the plants with functional FvTFL1 or silenced *FvFT1* develops into FM1 slower than in *fvtfl1* mutants, thus allowing formation of additional lateral IM2s.

In woodland strawberry, *FvTFL1* is highly expressed in the vegetative shoot apex and gradually downregulated during flowering induction (Koskela et al. 2012). Molecular antagonism between *TFL1* and *LFY* has been described as the mechanism controlling inflorescence architecture in Arabidopsis (Schultz and Haughn 1993, Prusinkiewicz et al. 2007). Subsequent studies in other species found that *AP1* may substitute *LFY* as *TFL1* antagonist in inflorescence development (Kobayashi et al. 2012; Ma et al. 2017). Here, we observed that the decrease of *FvTFL1* expression is associated with the progression of the vegetative SAM toward FM identity (Fig. 5D). The expression of the strawberry homologs of *LFY* and *AP1* showed the opposite pattern (Fig. 5, E and F). The expression of both *FvLFYa* (*FvH4_5g09660*) and *FvAP1* (*FvH4_6g29600*) began increasing in late IM1s, where *FvTFL1* was still present. *FvTFL1* expression was lowest in the FM tissues, coinciding with the highest expression of *FvLFYa* and *FvAP1*. Altogether, our data suggest that the role of TFL1 in maintaining IM identity (Alvarez et al. 1992; Schultz and Haughn 1993) is conserved in woodland strawberry.

To further elucidate the mechanism by which FvFT1 regulates inflorescence architecture in the absence of functional FvTFL1, we analyzed the expression levels of *FvTFL1, FvLFYa*, and *FvAP1* in the SAMs of *FIN56* (*FvTFL1*)*, Hawaii-4* (*fvtfl1*), and *FvFT1-RNAi fvtfl1* plants, respectively. We observed that the expression of *FvLFYa* and *FvAP1* was lower in the SAMs of *FIN56* and *FvFT1-RNAi* plants compared to *fvtfl1* (*Hawaii-4*) (Fig. 5, H and J). In addition, the expression of *FvTFL1* was the lowest in the *fvtfl1* mutant (Fig. 5G). Our results thus suggest that FvTFL1 and FvFT1 antagonistically control the transition to FM identity by regulating the expression of *FvLFYa* and *FvAP1*.

### A computational model points to heterochrony as the key determinant of the strawberry inflorescence diversity

**Figure 6.**
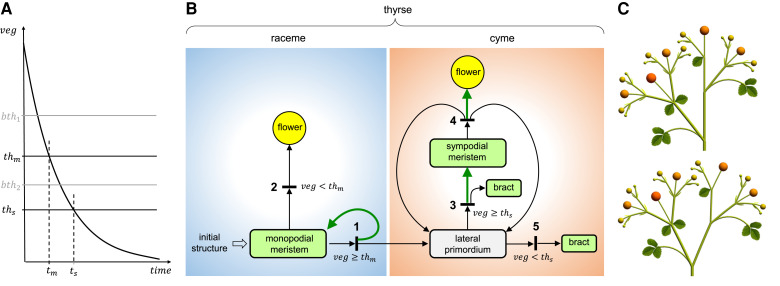
Developmental model of a woodland strawberry thyrse. **A)** Control of a thyrse development by vegetativeness (*veg*). Crossing threshold *th_m_* terminates the production of primordia by the primary (monopodial) meristem; crossing threshold *th_s_* terminates production of lateral primordia by the sympodial meristems. Times *t_m_* and *t_s_* at which these thresholds are crossed determine the inflorescence complexity (extent of branching). Additional thresholds, *bth_1_* and *bth_2_*, control the transitions of bracts from 3- to 1-lobed to narrow-scale forms. **B)** Petri net representation of the meristem production and fate. Circles and rectangles represent indicated plant organs. The term “lateral primordium” denotes an incipient structure yielding a lateral meristem supported by a bract, or a bract alone. Short black bars represent events taking place during development. These events are labeled by a number and are associated with conditions under which they may take place. Arcs with arrows represent relations between these events and the resulting structures. Two arrows originating in the same rectangle indicate alternative organ fates. Two or 3 arrows emanating from the same bar indicate production of multiple organs. Green-colored arrows indicate the introduction of an internode. **C)** The impact of the monopodial axis course on thyrse geometry. In the top model the monopodial axis is straight; in the bottom it deviates in the direction opposite to the lateral branch, as commonly observed in woodland strawberry (cf. Fig. 2, A, B, O, and P).

We constructed a parametrized computational model of the woodland strawberry inflorescence to show that the observed diversity of the inflorescence architecture can be attributed to the *FvTFL1-* and *FvFT1-*regulated rate of development. The model is expressed as an L-system, with separate rules capturing the monopodial development of the primary axis and the sympodial development of branches. Generic rules for both types of branching (cf. Prusinkiewicz and Lindenmayer 1990) are extended with a mechanism that controls transitions of individual meristems to flowers and terminates the formation of new meristems at the end of inflorescence development. These processes are controlled by a synthetic variable (integrating many influences) similar to vegetativeness (*veg*) (Prusinkiewicz et al. 2007), which is related to the concepts of a controller of phase switching (Schultz and Haughn 1993) and meristem maturation (Park et al. 2012). We illustrate the production and fate of meristems using a Petri net (Fig. 6B), the notion further described by Peterson (1981), and in application to plant modeling by Prusinkiewicz and Remphrey (2000). The use of Petri nets enhances the flowchart representation of inflorescence development proposed by Kellogg (2000) by explicitly representing events that produce multiple organs, which may develop concurrently with their parent meristem.

In the strawberry model, *veg* decreases monotonically with time (Fig. 6A). Its values are compared to 2 thresholds, *th_m_* and *th_s_*, which are pertinent to the fate of monopodial and sympodial meristems, respectively. As long as *veg ≥ th_m_*, the primary meristem periodically produces lateral primordia subtended by bracts (Event 1 in Fig. 6B). This process terminates when *veg* drops below *th_m_*, at which point the meristem produces a terminal flower (Event 2). A lateral primordium then develops into a lateral meristem supported by a bract (Event 3), which subsequently produces 2 next-order primordia and a terminal flower (Event 4). This process iterates periodically as long as *veg* ≥ *th_s_*, giving rise to a dichasial cyme. Eventually *veg* drops below *th_s_*, which results in the production of bracts without associated meristems (Event 5) and arrests further branching. The distinct development of branches originating at the same node, in particular the case when only 1 lateral branch is present, are captured by introducing a small difference in the value of threshold *th_s_* between sibling meristems. This difference may be related to the unequal space available for the development of primordia on opposite sides of an elongated meristem (Fig. 2Q).

**Figure 7.**
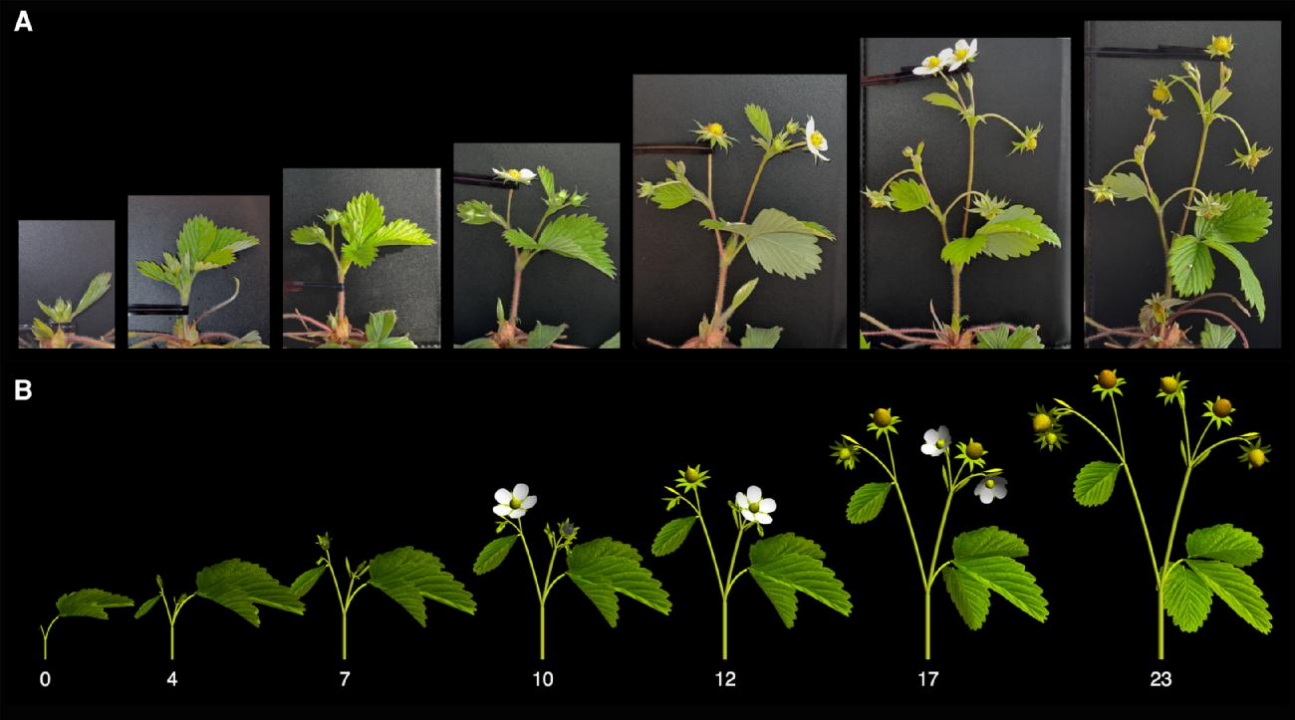
Development of a woodland strawberry inflorescence. **A)** Snapshots of inflorescence growth in a WT (GER12 × FIN2) woodland strawberry. Photographs were taken with the inflorescence supported in a vertical position to expose the branching pattern. Flowering was induced by growing plants at 11 °C under a 12 h/12 h (day/night) photoperiod for 6 wk, then moved to 17 °C and a 18 h/6 h photoperiod during inflorescence growth. Numbers indicate days of observation. **B)** Simulated development calibrated to sequence A.

In addition to the thresholds controlling branching architecture, the model includes thresholds *bth_1_* and *bth_2_* (Fig. 6A), which control the transition of bracts from the 3-lobed form to 1-lobed form and to narrow scales, respectively. The model also includes several parameters and growth functions that control the geometry of the phenotypes of interest. Parameter values and functions were calibrated such that the simulated inflorescence development (Movie 1) closely approximates observations (Fig. 7), including the deviation of the primary axis from the straight course (Fig. 6C). For details, see the supplemental model code, model description, and the parameter values listed in Supplemental Table S1.

**Figure 8.**
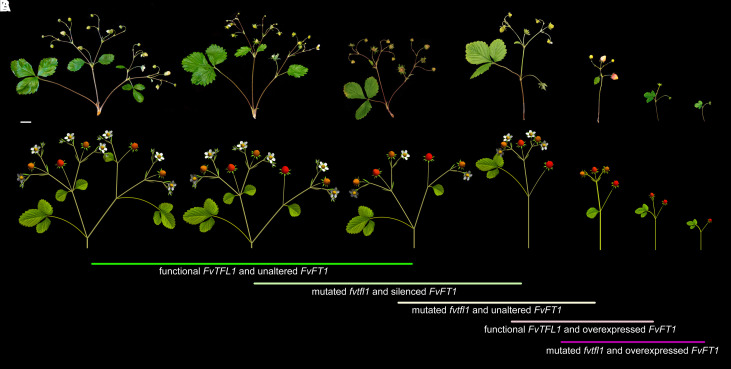
Diversity of woodland strawberry inflorescence phenotypes can be reproduced by altering model parameters. **A)** Observed diversity of inflorescence phenotypes. Scale bar equals 1 cm. **B)** Phenotypes reproduced by the model. Lines with text describe the genetic constitution of the plants. The color of the line indicates the rate of *veg* decline (green, slower; magenta, faster).

We experimented with the model to capture the key inflorescence features of the plants with different levels of *FvTFL1* and *FvFT1*. The most prominent trait that distinguishes the architectures collected in Fig. 8A are the gradual decrease of the maximum order of branching, caused by an accelerated switch of IMs to the flowering state. This decrease is accompanied by the accelerated progression of bracts from the 3-lobed leaf-like form at the most basal position to the 1-lobed and small-scale forms at more distal positions. Both the branching architecture and bract form are controlled in the model by the initial value and rate of decline of *veg*, and the thresholds *th_m_*, *th_s_*, *bth_1_*, and *bth_2_* with respect to *veg* (Fig. 6A). We observed that the sequence of inflorescence architectures (branching topologies) presented in Fig. 8A can be reproduced simply by gradually increasing the rate of decline of *veg*, with minimal adjustments to other parameters except for the extreme phenotype of the *FvFT1-OX fvtfl1* plants (Fig. 8B and Movies 2 to 8; Supplemental Table S1). A similar effect is caused by gradually reducing the initial value of *veg*. These results support the conclusion that the diversity of strawberry inflorescences is a manifestation of heterochrony, in which *FvTFL1* decelerates, and *FvFT1* accelerates the rate of *veg* decline, respectively.

## Discussion

Almost a century ago, Darrow (1929) documented the extensive variability of inflorescence architectures in strawberry. This variability has led to inconsistent characterizations of the inflorescence architecture, classified mostly as variants of cymes, but also as corymbs. We have shown that the inflorescence of woodland strawberry is a determinate thyrse and analyzed the regulation of its development and variability using a computational model informed by microscopic and molecular data.

### Two types of IMs produce strawberry thyrse

According to the ontogenic view of inflorescence diversity, distinct inflorescence architectures are produced by meristems of different type (Claßen-Bockhoff and Bull-Hereñu 2013). Our data show that the determinate thyrse of woodland strawberry—a sequence of cymes supported by a monopodial axis—is produced by IMs with distinct geometries. As in Arabidopsis, the primary meristem is dome-shaped, which promotes sequential production of lateral meristems, characteristic of racemes. In contrast to the indeterminate racemes of Arabidopsis, however, IM1 of a typical strawberry inflorescence acquires flower identity after initiating 2 to 3 branches, thus forming a closed monopodial axis. Due to the relatively large size of IM2s, the main axis changes its course at each branching point, which obscures its monopodial character. We attribute previous misclassifications of the strawberry inflorescences to this phenomenon.

In contrast to IM1, each IM2 has a crescent-shaped cross-section. Such differences in geometries of the reproductive meristems were not reported in the previously studied systems, including tomato or pea, in which the shapes of the primary and secondary meristems are the same (Berbel et al. 2012; Park et al. 2012). According to the first available space theory (Hofmeister 1868), new organ primordia are initiated in the largest available space between the previous ones. The crescent shape of the lateral IMs promotes concurrent initiation of next-order bracts and branches in pairs situated near the opposite poles of their parent meristem, which leads to the typical cymose branching. The cessation of branching is often preceded by a reduction of this pattern, with only a single branch developing at some or all of the highest-order branching points. We hypothesize that the other branch does not develop due to a space restriction caused by a higher proximity to the inflorescence center.

Superimposed on these geometric factors are processes controlling the fate of meristems: the switch of the vegetative to the flowering state, and the cessation of the production of lateral meristems. In general, these processes can be attributed to 2 different mechanisms. One possibility is that, upon producing a lateral primordium, the primary meristem, the lateral primordium, or both, permanently acquire an identity different from that of their parent. The type of generated inflorescence is fixed by the structure of these transitions. For instance, a raceme will result if the lateral primordia acquire flowering identity while the main meristem remains vegetative, the opposite assignment of identities will result in a cyme, and a combination of both models can produce a thyrse (Prusinkiewicz and Lindenmayer 1990). Another possibility is that the difference between lateral and main meristems is transient. A wide spectrum of inflorescence types can be produced by a model in which newly created lateral meristems have a different vegetativeness from their parents, but this difference is reversed when these lateral meristems start producing next-order primordia on their own (Prusinkiewicz et al. 2007).

The classes of inflorescences generated by the “permanent” and “transient” models overlap to some extent, which leads to the question of which model fits the data better when both models are possible. For instance, in the case of Arabidopsis, inflorescences with different expression levels of *TFL1* and *LFY* represent a continuum of branching forms ranging from single flowers to simple and compound racemes to panicles. This continuum is readily captured by changing parameters of the transient model (Prusinkiewicz et al. 2007). The observed inflorescences of the woodland strawberry, however, have a relatively rigid structure: the main axis is monopodial, the lateral branches are cymose. For this reason, although thyrses can also be produced by the transient model, the presented model has been constructed under the more rigid assumption that changes in the meristem states are permanent. With different rates of vegetativeness decline, and some adjustments of threshold values, the model robustly reproduces the observed range of strawberry inflorescences without generating architectures that we have not observed, and thus provides a convenient conceptual framework for interpreting experimental data. Nevertheless, it would be interesting to see whether more extreme changes in the expression of *FvTFL1* and *FvFT1* would produce architectures beyond the thyrse template.

### FvTFL1 and FvFT1 antagonistically regulate the complexity of woodland strawberry inflorescences

Previous studies uncovered the role of *FvTFL1* in the regulation of flowering time in woodland strawberry (Koskela et al. 2012, 2017). Our results show that *FvTFL1* also regulates inflorescence development and architecture. *FvTFL1* expression pattern in woodland strawberry differs from the previously studied species with simple and compound inflorescence architectures. Our results show that as the SAM progresses toward the IM and then to the FM state, *FvTFL1* expression gradually decreases. While in monopodial Arabidopsis *TFL1* expression is upregulated during SAM to IM transition (Alvarez et al. 1992; Schultz and Haughn 1993), in woodland strawberry it is downregulated. Similar to Arabidopsis, upregulation of *DET*, a *TFL1* homolog of pea, confers the primary IM identity in a compound raceme (Foucher et al. 2003). *TFL1* expression pattern in woodland strawberry differs also from cymose tomato, where *TFL1* (*SP*) is not expressed in IMs and does not contribute to inflorescence architecture (Pnueli et al. 1998; Thouet et al. 2008; Park et al. 2012). During the transition from IM1 to FM, we observed further downregulation of *FvTFL1* accompanied by the activation of *FvLFYa* and *FvAP1*, consequently ceasing the formation of additional IM2s. Together with the previous experiments (Koskela et al. 2012, 2017), our data suggest a dual role for FvTFL1 in maintaining the identities of SAM and IM in a dosage dependent manner.

Although it is determinate, the primary IM (IM1) of woodland strawberry is related to Arabidopsis in the sense that the loss of functional FvTFL1 accelerates the formation of the terminal flower (Alvarez et al. 1992; Fig. 5, A and B). This results in early cessation of the lateral meristem (IM2) initiation. In the *fvtfl1* mutant, only 1 lateral branch is formed on the primary monopodial inflorescence axis, and the number of branching iterations is reduced. This reduction of inflorescence complexity results from the overall decrease of vegetativeness in the *fvtfl1* plants as indicated by the presence of *FvLFYa* and *FvAP1* transcripts already in the SAM. However, despite the reduced number of branching iterations, the dichasial branching pattern of the lateral axes is maintained. This is due to the unaltered crescent shape of lateral meristems that allows nearly simultaneous initiation of new lateral IMs.

Mutations of *FT* genes have been shown to increase the complexity of both monopodial and sympodial inflorescences (Park et al. 2012; Lee et al. 2019). This function is conserved in woodland strawberry. Silencing of *FvFT1* partially compensated for the lack of functional FvTFL1 and reduced the expression of *FvLFYa* and *FvAP1* in the SAMs. As a result, the percentage of inflorescences with 2 lateral branches on the primary axis increased in *FvFT1*-RNAi plants. Moreover, the number of branching iterations was increased. In contrast, ectopic *FvFT1* expression resulted in inflorescences with only a few flowers. Thus, by manipulating the expression of *FvFT1* we can either enhance or complement the effect of *fvtfl1* mutation. The observed antagonism of FvTFL1 and FvFT1 is consistent with findings that FT and TFL1 proteins competitively bind to bZIP transcription factor FD and 14-3-3 proteins to activate or repress, respectively, the expression of *LFY* and *AP1* (Kaneko-Suzuki et al. 2018; Zhu et al. 2021).

In conclusion, our data highlight the difference in the identities of the primary and higher order meristems. This suggests the presence of an additional factor required to define their identities, thus creating a thyrse framework in which FvTFL1 and FvFT1 operate. Identification of this factor and uncovering its potential role in the regulation of FvTFL1/FvFT1 antagonism is crucial to establish a detailed molecular mechanism of thyrse development. This is agriculturally important because inflorescence architecture, referring to the complexity of branching patterns and final numbers of flowers, is associated with crop yield. Moreover, in cultivated strawberry, the final berry size depends on its position in the inflorescence (Webb et al. 1974). Our experimental results and model provide a stepping stone for further work to optimize berry yield by modifying the expression of cultivated strawberry *TFL1* and *FT1* homologs.

## Materials and methods

### Plant material

Nine *F. vesca* and 7 *F. vesca semperflorens* (*fvtfl1* mutant) accessions were used in this study (Supplemental Table S2). Previously reported *FvFT1* overexpression (OX) and RNA silencing (RNAi) lines in *fvtfl1* (*Hawaii-4*) background were used (Koskela et al. 2012; Rantanen et al. 2014). The specificity of the RNAi construct to *FvFT1* was confirmed by analyzing the expression of *FvFT2* and *FvFT3* in the FM tissues (Supplemental Fig. S6, A and B). To obtain *FvFT1-OX* plants in *FvTFL1* background, we crossed *FvFT1-OX5 (Hawaii-4)* line with *FvTFL1* accession *(FIN56)* and self-pollinated the progeny. F_2_ plants carrying FIN56 *FvTFL1* allele and the desired *FvFT1* overexpression construct were used in the experiments.

### Growth conditions and phenotyping

All plants were grown in greenhouse conditions with controlled temperature and photoperiod. In the greenhouse, the plants were illuminated with 150 *µ*mol m^−2^ s^−1^ light intensity using high-pressure sodium lamps (Airam 400W, Kerava, Finland). Germinated from seeds or clonally propagated plants were first potted into 7 × 7 cm plastic pots filled with peat moss (Kekkilä, Finland) and kept in the greenhouse under plastic covers for 2 wk. Plants were pregrown at 18 °C and an 18 h photoperiod for 4 to 6 wk, and periodically supplemented with fertilizer (NPK 17-4-25; Kekkilä). Plants were then potted into 13 cm Ø pots and grown until the inflorescences were fully formed. Plants were irrigated with tap water supplemented with fertilizer (NPK 17-4-25; Kekkilä).

For the analysis of FvFT1 and FvTFL1 functions, we pregrew 8 to 13 seed germinated plants per transgenic line or cultivar, or 6 to 13 clonally propagated plants per accession with functional FvTFL1, in growth rooms under an 18 h/6 h (day/night) photoperiod (AP67, Valoya, Finland) and 25 °C temperature. For floral induction, the plants were then moved to a 12 h/12 h (day/night) photoperiod at 18 °C in the greenhouse for 6 wk. After the treatment, the plants were kept under 18 °C and an 18 h/6 h (day/night) photoperiod until the inflorescences were fully developed. Inflorescence architecture was analyzed by counting the total number of flowers and flower buds, the number of branching iterations in the longest branching path, and the number of branches on the primary axis. For the functional analysis of FvTFL1, the first fully developed inflorescence representing a biological replicate was analyzed from each plant. For the functional analysis of FvFT1, 5 fully developed inflorescences per plant were analyzed.

### Genotyping

DNA was extracted from young, folded leaves of *fvtfl1* mutants and WT plants listed in Supplemental Table S2 as in Koskela et al. (2012). PCR amplified *FvTFL1* fragments were excised from agarose gel, purified using gel purification kit (K0832, Thermo Scientific, Waltham, USA), and sequenced using primers listed in Supplemental Table S3.

### RNA extraction and RT-qPCR analysis

Meristems were dissected under stereomicroscope (Zeiss Stemi 2000). SAMs, IMs, and FMs were excised and collected into separate Eppendorf tubes. Five to 10 meristems were pooled into 1 tube representing a biological replicate and used for RNA extraction. Total RNA was extracted from pooled meristem samples as in Mouhu et al. (2009) and treated with rDNase (Macherey-Nagel GmbH, Düren, Germany). Five hundred nanograms of total RNA were used for cDNA synthesis using ProtoScriptII reverse transcriptase. RT-qPCR was performed using a LightCycler 480 SYBR Green I Master kit (Roche Diagnostics, Indianapolis, USA) and a Roche LightCycler (Roche Diagnostics) with 3 technical replicates for each of the tested genes. *FvMSI1 (FvH4_7g08380)* was used as a reference gene. The qPCR primers used in this study are listed in Supplemental Table S3.

### SEM imaging

Dissected meristem samples were fixed in an FAA buffer (3.7% v/v formaldehyde 5% v/v acetic acid, and 50% v/v ethanol) overnight and transferred through ethanol series (50%, 60%, 70%, 80%, 90%, 100%, and 100%) under mild vacuum (∼0.6 atm). Critical point drying was done using a Leica EM CPD300 (Leica Mikrosystems GmbH, Vienna, Austria). The dried samples were then coated with platinum by a Quorum Q150TS coater (Quorum Technologies, UK). The samples were examined under a Quanta 250 FEG (FEI, Hillsboro, OR, USA) scanning electron microscope located at the Electron Microscopy Unit (Institute of Biotechnology, University of Helsinki). Pseudocoloring was done in Adobe Photoshop CC 2019.

### Statistical analyses

The phenotypic data (flowering time, number of flowers per inflorescence and branching iterations) were analyzed using random intercept models, *y* = *β*_0_ + *β*_1_*X* + *Gt* + *ɛ*, where *y* = dependent variable, *β*s = fixed effects, *X* = design matrices for *FvTFL1* background or *FvFT1* transgenic construct, *Gt* = random accession or independent line effect, and *ɛ* = an error term. Poisson distribution model was applied for count data. Statistical analyses were performed using the R/lme4 package (Bates et al. 2015). All analyses were performed in R version 4.1.1 (R Core Team 2021).

### Inflorescence modeling

The models were written in the L-system-based L + C plant modeling language (Karwowski and Prusinkiewicz 2003) and executed using the lpfg simulator incorporated into the Virtual Laboratory (vlab) v5.0 plant modeling software (algorithmicbotany.org/virtual_laboratory or github.com/AlgorithmicBotany/vlab). All simulations were performed on MacBook Pro computers under macOS High Sierra 10.13.6. The supplemental materials include 3 complete vlab objects (Supplemental Files S2 to S4), which are versions of the model: the basic version with a simplified representation of plant organs (bracts and flowers), convenient for analyzing the model logic; the extended version with full representation of organs, used in the model calibration shown in Fig. 7; and the same extended version configured to simulate the diverse phenotypes shown in Fig. 8. The key elements of the model code are discussed in the supplemental model description (Supplemental File S1). Parameters used to simulate the diverse phenotypes were found by interactively exploring the model parameter space using the tools included in vlab (control panels and graphically defined timelines and functions). The reference model (Fig. 7) was calibrated using the method described by Cieslak et al. (2022).

### Accession numbers

*FvTFL1 FvH4_6g18480*, *FvFT1 FvH4_6g00090*, *FvLFYa FvH4_5g09660*, *FvAP1 FvH4_6g29600*, *FvMSI1 FvH4_7g08380*, *FvFT2 FvH4_4g30710*, and *FvFT3 FvH4_3g09870*.

## Supplementary Material

koad086_Supplementary_Data

## Supplemental data

The following materials are available in the online version of this article.

**Supplemental Figure S1.** Inflorescence phenotypes of 7 *fvtfl1* and 9 *FvTFL1* genotypes.

**Supplemental Figure S2.** Alignment of the part of the first exon of *FvTFL1* gene in 7 cultivars and 9 WT accessions.

**Supplemental Figure S3.** *FvFT1* expression levels in the leaves of *Hawaii-4* (control), *FvFT1* overexpression (OX), and *FvFT1* silencing (RNAi) transgenic lines.

**Supplemental Figure S4.** *FvFT1* overexpression in the *fvtfl1 (Hawaii-4)* background induces flowering in all axillary buds and reduces overall plant size.

**Supplemental Figure S5.** *FvFT1* overexpression in the functional *FvTFL1* background.

**Supplemental Figure S6.** The expression of *FvFT2* and *FvFT3* in the FMs of *Hawaii-4* and *FT1-RNAi6* plants.

**Supplemental Table S1.** Model parameters used to produce inflorescences in Fig. 7 and Movie 1 (Phenotype 0); Fig. 8 and Movies 2 to 8 (Phenotypes 1 to 7).

**Supplemental Table S2.** Plant material used in this study.

**Supplemental Table S3.** Primers used in this study.

**Supplemental Data S1.** Summary of the statistical analyses.

**Supplemental File S1.** Supplemental model description.

**Supplemental File S2.** Parametrized L-system model of the woodland strawberry thyrse architecture “strawberry-basic,” using schematic visualization of flowers and fruits (Phenotypes 0 to 7).

**Supplemental File S3.** Parametrized L-system model of the woodland strawberry thyrse architecture “strawberry-calibrating,” illustrating model calibration to the developmental sequence of the GER12 × FIN2 cross (Phenotype 0) shown in Fig. 7.

**Supplemental File S4.** Parametrized L-system model of the woodland strawberry thyrse architecture “strawberry-diversity,” generating Movies 2 to 8 and the snapshots shown in Fig. 8 (Phenotypes 1 to 7).
