# Supplementary material for: Diversity of woodland strawberry inflorescences arises from heterochrony regulated by TERMINAL FLOWER 1 and FLOWERING LOCUS T
Source: Plant Cell. 2023 Mar 21;35(6):2079–94. doi: 10.1093/plcell/koad086 (PMC10263268; doi:10.1093/plcell/koad086)
Supplement: koad086_Supplementary_Data [file koad086_Supplementary_Data.zip › Supplemental Movie Legends.docx]

**Supplemental Movie Legends**

**Supplemental Movie S1.** Simulated WT woodland strawberry development calibrated to the GER12 x FIN2 cross (Phenotype 0).

**Supplemental Movie S2.** Simulated development of the inflorescence Phenotype 1 shown in Figure 8.

**Supplemental Movie S3.** Simulated development of the inflorescence Phenotype 2 shown in Figure 8.

**Supplemental Movie S4.** Simulated development of the inflorescence Phenotype 3 shown in Figure 8.

**Supplemental Movie S5.** Simulated development of the inflorescence Phenotype 4 shown in Figure 8.

**Supplemental Movie S6.** Simulated development of the inflorescence Phenotype 5 shown in Figure 8.

**Supplemental Movie S7.** Simulated development of the inflorescence Phenotype 6 shown in Figure 8.

**Supplemental Movie S8.** Simulated development of the inflorescence Phenotype 7 shown in Figure 8.
