# Supplementary material for: Diversity of woodland strawberry inflorescences arises from heterochrony regulated by TERMINAL FLOWER 1 and FLOWERING LOCUS T
Source: Plant Cell. 2023 Mar 21;35(6):2079–94. doi: 10.1093/plcell/koad086 (PMC10263268; doi:10.1093/plcell/koad086)
Supplement: koad086_Supplementary_Data [file koad086_Supplementary_Data.zip › Supplemental_Inflormation.pdf]

Figures

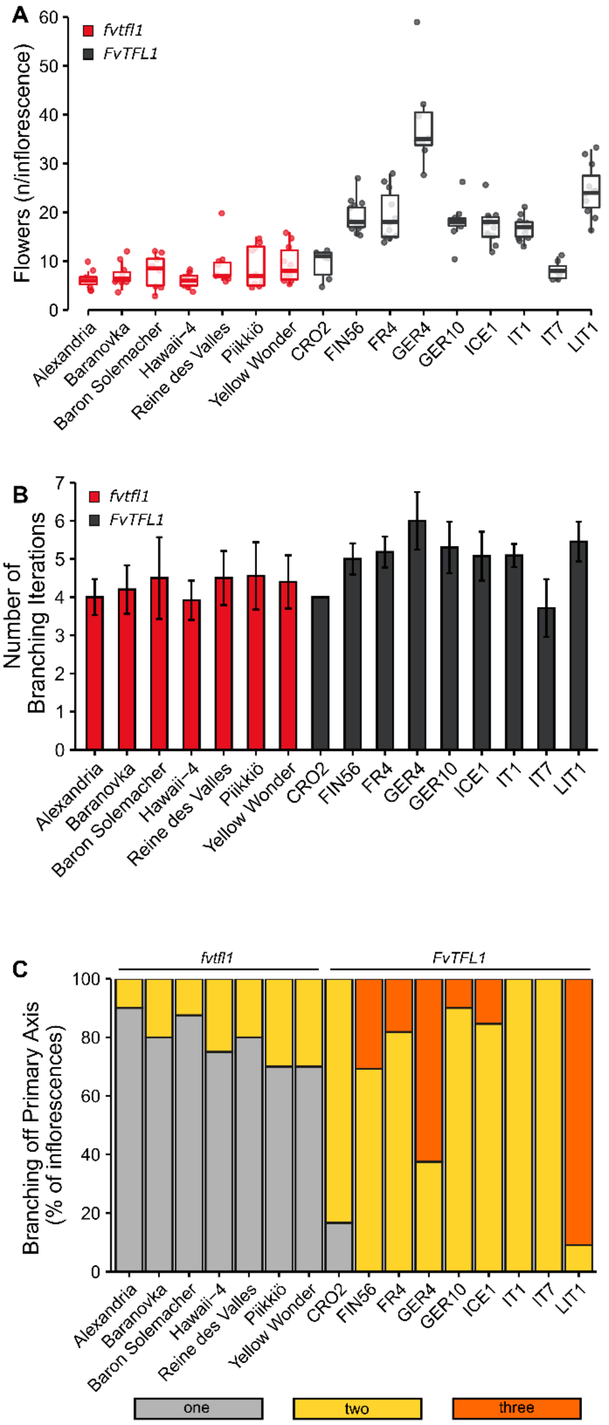

Supplemental Figure S1. Inflorescence phenotypes of seven *fvtfl1* and nine *FvTFL1* genotypes. Supports Figure 3. **A.** Number of flowers on the first emerged fully-developed inflorescence per plant (n = 6 – 13). Boxplots and points show distribution of raw data. Each point represents an individual inflorescence. **B.** Average number of branching iterations along the longest branching path. Bars and error bars represent the mean ± standard deviation (n = 6 – 13). **C.** Percentage of observed inflorescences with one, two, or three branches on the primary branching axis.

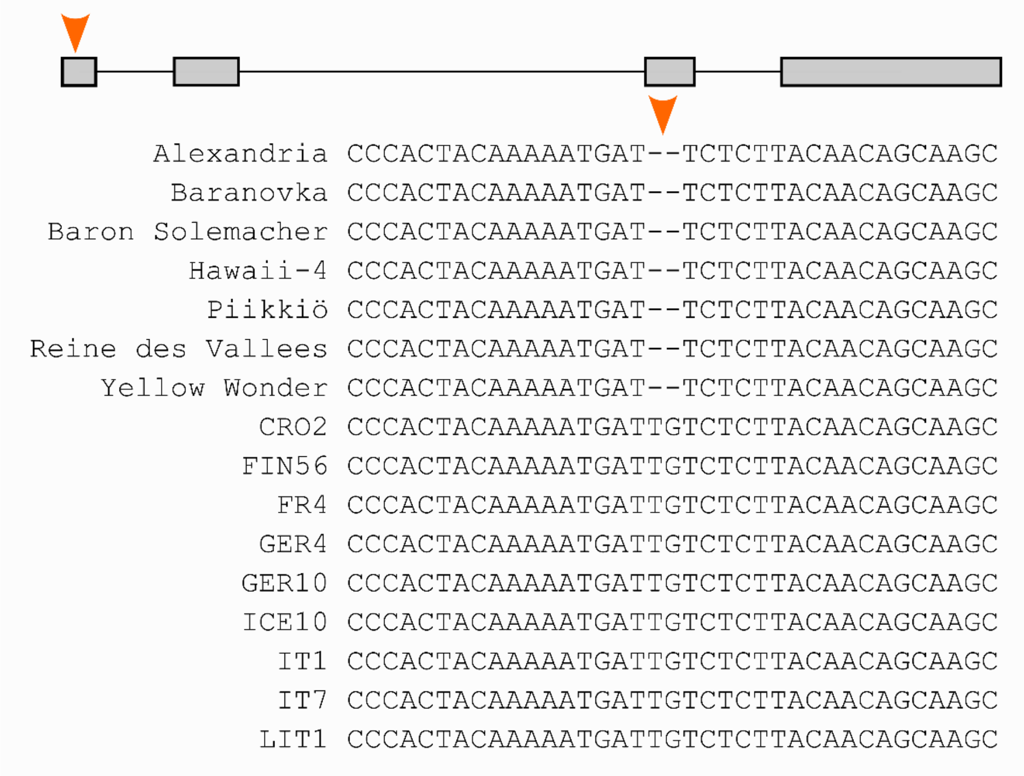

Supplemental Figure S2. Alignment of the part of the first exon of *FvTFL1* gene in seven cultivars and nine WT accessions. Supports Figure 3.

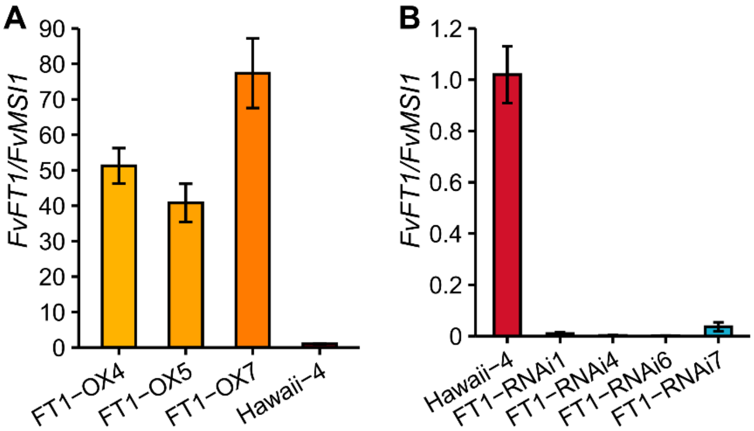

Supplemental Figure S3. *FvFT1* expression levels in the leaves of *Hawaii-4* (control), *FvFT1* overexpression (OX) and *FvFT1* silencing (RNAi) transgenic lines. Supports Figure 4. Error bars and show standard error (n = 3 – 4). Biological replicates represent 1cm Ø leaf disc excised from a plant at ZT16. Expression levels were normalized to *Hawaii-4*. *FvMSI1* was used as a reference gene.

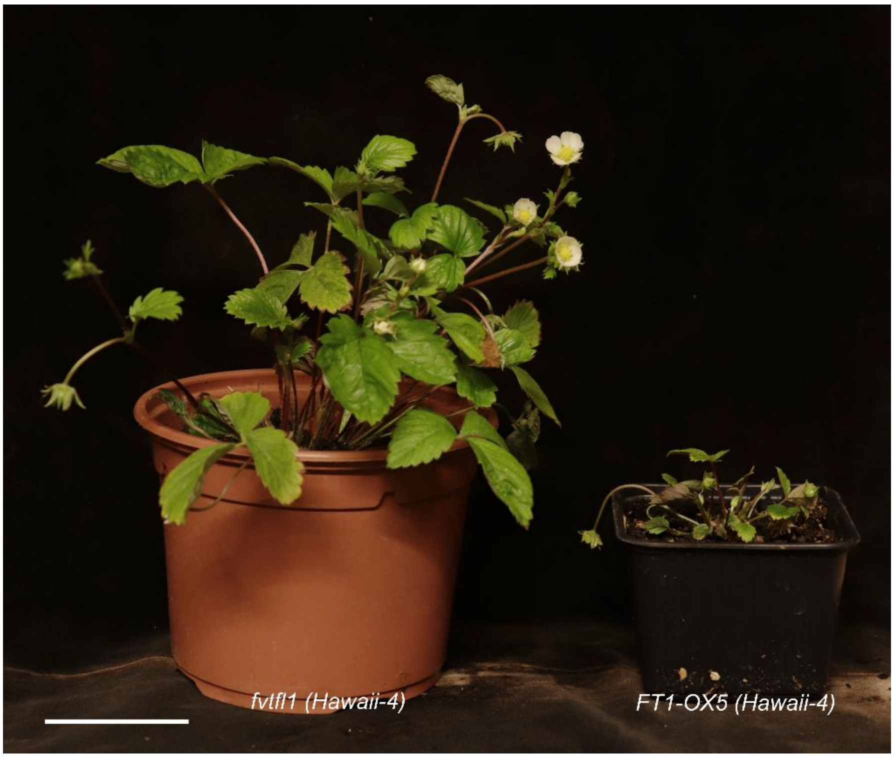

Supplemental Figure S4. *FvFT1* overexpression in the *fvtfl1 (Hawaii-4)* background induces flowering in all axillary buds and reduces overall plant size. Supports Figure 4. Scale bar equals 5 cm.

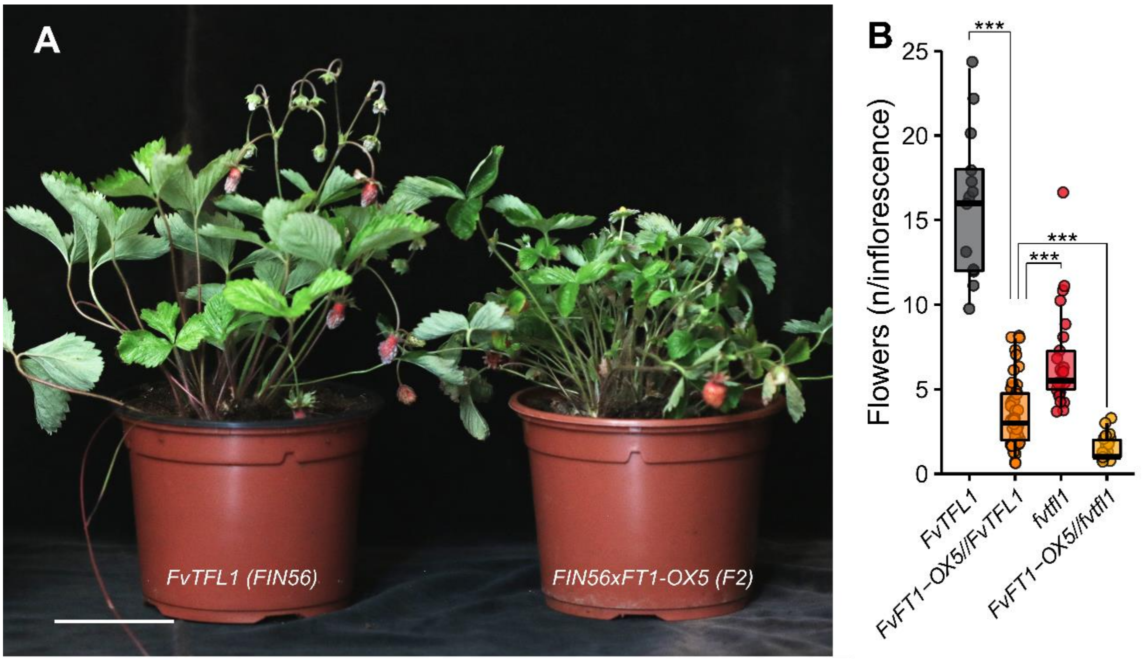

Supplemental Figure S5. *FvFT1* overexpression in the functional *FvTFL1* background. Supports Figure 4. **A.** Overall plant phenotypes. **B.** The number of flowers per inflorescence in *FvFT1* overexpression plants in functional and non-functional *FvTFL1* backgrounds. Boxplots and points show the distribution of raw data. Each point represents an individual inflorescence (n = 16 – 54). Up to six inflorescences per plant were examined. Data were analyzed by fitting a generalized linear model (GLM). *** indicate P value < 0.0001 between the groups (Tukey's HSD).

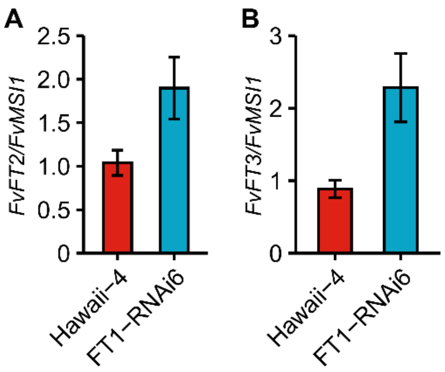

Supplemental Figure S6. The expression of *FvFT2* **(A)** and *FvFT3* **(B)** in the FMs of *Hawaii-4* and *FT1-RNAi6* plants. Supports Figure 4. Bars and error bars show mean ± standard error (n = 3 – 4). Expression levels were normalized to *Hawaii-4*.

Tables

Supplemental Table S1. Model parameters used to produce inflorescences in Figure 7 (Phenotype 0) and Figure 8 (Phenotypes 1-7). Parameters shown in bold are discussed in the main text or Supplemental Model Description; the remaining parameters are explained in the code. Only values differing from the previous column are shown. The key parameter (in red) differentiating the models is `veg_half_life`, which determines the time (measured in plastochrons) over which *veg* decreases to ½ of its initial value. Related parameters are grouped together as in the code.

**Phenotype**	**0**	**1**	**2**	**3**	**4**	**5**	**6**	**7**
max_age	8							
**dt**	0.02							
**plastochron**	1							
**delay_mono**	0.44							
**delay_sym**	0.3	0						
**delay_diff**								
**veg_init**	1							
**veg_half_life**	1.98	2.77	2.42	2.07	1.64	1.37	1.05	1.05
**th_m**	0.43							
**th_s**	0.27				0.23			0.6
**th_diff**	0.08				0			
b_th_1	0.6						0.48	
b_th_2	0.45							
pleiotropic_scale	4	1				0.82	0.5	0.4
peduncle_length_ctrl	0.4	0.2		0.3	0.65	0.7	0.75	0.6
int_len_power	1.2							
internode_segments	10							
**distal_diameter**	0.011				0.008	0.011		
**pipe_model_power**	3							
**angle_main**	12	35	38	17	27			35
**angle_lateral**	10	17	13	29	7			17
**angle_sympodial**	20	32			35		32	
int_elasticity	0.32	0.06						
int_elasticity_power	1.3							
bract_angle_branch	5							
bract_angle_no_branch	25							17
ang_adjustment_1	0						6	14
ang_adjustment_2	35							
petiole_length_1	0.2	0.7					0.3	0.3
petiole_length_2	0.02							
petiole_elasticity_1	1.8							
petiole_elasticity_2	1							
bract_size_power	0.5							

Supplemental Table S2. Plant material used in this study.

**Accession Name**	**Origin/Collection Site**
Alexandria (*PI602923*)	NCGR*
Baranovka	Lyubov Kuznetsova**
Baron Solemacher (*PI551507*)	NCGR*
Hawaii-4 (*PI551572*)	NCGR*
Piikkiö	Unknown
Reine des Vallees (*PI551824*)	Dr. Amparo Monfort***
Yellow Wonder (*PI551827*)	NCGR*
CRO2	44°46’15.1896’’ (N) 15°38’51.6408’’ (E)
FIN56 (*PI551792*)	NCGR*
FR4	48°25’24.4452’’ (N) 7°39’48.0168’’ (E)
GER4	50°59’6.9036’’ (N) 11°19’21.216’’(E)
GER10	47°33’21.168’’ (N) 10°1’17.2632’’ (E)
ICE10	64°45’36.774’’ (N) -21°35’35.2824’’ (E)
IT1	45°56’13.362’’ (N) 10°48’20.2392’’ (E)
IT7	46°16’30.1188’’ (N) 11°16’54.9372’’ (E)
LIT1	54°47’51.7812’’ (N) 25°20’54.2292’’ (E)

*National Clonal Germplasm Repository, Corvallis, USA; ** The Federal Research Center Institute of Cytology and Genetics, Novosibirsk, Russia; *** Centre for Research in Agricultural Genomics, Barcelona, Spain

Supplemental Table S3. Primers used in this study.

Name	Forward	Reverse	Reference
FvTFL1 (FvH4_6g18480) (cloning/sequencing)	ATGGCAAGAATGTCGGAACC	CTAGCGTCTTCTTGCTGCC	
FvTFL1 (FvH4_6g18480) (RT-qPCR)	AACGGCAGCAACAGGAAC	CTGGCACCACAGATGCTACA	Koskela et al., 2012
FvAP1 (FvH4_4g29600) (RT-qPCR)	AGCTCAGGAGGTTCATGACTG	TAAGGTCGAGCTGGTTCCTC	Koskela et al., 2012
FvMSI1 (FvH4_7g08380) (RT-qPCR)	TCTCCACACCTTTGATTGCCA	ACACCATCAGTCTCCTGCCAAG	Mouhu et al., 2009
FvFT1 (FvH4_6g00090) (RT-qPCR)	CAATCTCTTGGCCGAAAACT	TGAGCTCAAACCTTCCCAAG	Koskela et al., 2012
FvLFYa (FvH4_5g09660) (RT-qPCR)	GATGACAGAATCAATGGAGGAG	CTGGTTTGTGACCTTGGTGG	
FvFT2 (FvH4_4g30710) (RT-qPCR)	ACTCGGTGGCTTGTGTTTTC	ATCACTCTCCCGACGACAAG	Nakano et al., 2015
FvFT3 (FvH4_3g09870) (RT-qPCR)	AGCCGTTCACCAAGTCTGTG	GTGGACAACATGAGAAGGTTTG	Nakano et al., 2015
