# Supplementary material for: Diversity of woodland strawberry inflorescences arises from heterochrony regulated by TERMINAL FLOWER 1 and FLOWERING LOCUS T
Source: Plant Cell. 2023 Mar 21;35(6):2079–94. doi: 10.1093/plcell/koad086 (PMC10263268; doi:10.1093/plcell/koad086)
Supplement: koad086_Supplementary_Data [file tpc22.01077.PeerReviewReport.pdf › tpc22.01077.PeerReviewReport.pdf]

Diversity of woodland strawberry inflorescences arises from heterochrony regulated by *TERMINAL FLOWER 1* and *FLOWERING LOCUS T*

Sergei Lembinen, Mikolaj Cieslak, Teng Zhang, Kathryn Mackenzie, Paula Elomaa, Przemyslaw Prusinkiewicz, Timo Hytönen

Corresponding authors: Timo Hytönen: timo.hytonen@helsinki.fi, Przemyslaw Prusinkiewicz: pwp@ucalgary.ca

Review timeline:

**TPC2022-RA-001077**	Submission received:	Nov. 4, 2022
	1st Decision:	Dec. 29, 2022 *revision requested*
**TPC2022-RA-001077R1**	1st Revision received:	Feb. 1, 2023
	2nd Decision:	Feb. 26, 2023 *acceptance pending, sent to science editor*
	Final acceptance:	Mar. 14, 2023

REPORT: (The report shows the major requests for revision and author responses. Minor comments for revision and miscellaneous correspondence are not included. The original format may not be reflected in this compilation, but the reviewer comments and author responses are not edited, except to correct minor typographical or spelling errors that could be a source of ambiguity.)

TPC2022-RA-01077 1st Editorial decision – *revision requested* Dec. 29, 2022

We have received reviews of your manuscript entitled "Diversity of the woodland strawberry inflorescences results from heterochrony antagonistically regulated by FvTFL1 and FvFT1." Thank you for submitting your best work to The Plant Cell. The editorial board agrees that the work you describe is substantive, falls within the scope of the journal, and may become acceptable for publication, pending revision and potential re-review.

We ask you to pay attention to the following points in preparing your revision:

Both reviewers find your work well done and well presented, however there is a difference in opinion as to its suitability for the journal. Reviewer 1 finds it lacks novel mechanistic insight and is another example of the previously described antagonism between TFL1 and FT in controlling inflorescence architecture. Reviewer 2 finds that applying this model to another, complex inflorescence architecture is sufficient for the journal and that the paper needs no changes for acceptance. Based on the comments of the reviewers and the editorial consultation, we would like to request a revision of the manuscript. We are not requesting any additional data, however we would ask that you edit the Abstract, Introduction and Discussion to emphasize more clearly the novel aspects of your manuscript in defining the Fragaria inflorescence architecture and the effectiveness of the FT/TFL antagonism model in explaining this complex architecture. Try to make clear with these changes the unexpected aspects of your results, and thereby answer the reservations of reviewer 1 concerning lack of novelty.

-- Reviewer comments:

TPC2022-RA-01077R1 1st Revision received Feb. 1, 2023

Reviewer comments and **author responses**:

<u>Comments from the editorial board:</u>

Both reviewers find your work well done and well presented, however there is a difference in opinion as to its suitability for the journal. Reviewer 1 finds it lacks novel mechanistic insight and is another example of the previously described antagonism between TFL1 and FT in controlling inflorescence architecture. Reviewer 2 finds that applying this model to another, complex inflorescence architecture is sufficient for the journal and that the paper needs no changes for acceptance. Based on the comments of the reviewers and the editorial consultation, we would like to

request a revision of the manuscript. We are not requesting any additional data, however we would ask that you edit the Abstract, Introduction and Discussion to emphasize more clearly the novel aspects of your manuscript in defining the Fragaria inflorescence architecture and the effectiveness of the FT/TFL antagonism model in explaining this complex architecture. Try to make clear with these changes the unexpected aspects of your results, and thereby answer the reservations of reviewer 1 concerning lack of novelty.

RESPONSE: We have now revised the abstract, introduction as well as the discussion and emphasized the following new/novel aspects in our results:

- **Given the long history of characterizations of the strawberry inflorescence, we were surprised to find that it has a fundamentally different architecture than previously thought, i.e., is a thyrse, produced by two distinct types of IM. This discovery provided the key to explain the observed diversity of the strawberry inflorescence architectures. Unexpectedly, we found that all the observed inflorescence architectures follow a common template – a thyrse. In spite of varied appearances (amplified by the erratic course of the main axis), the differences are limited to the degree of branching. With a combination of racemose primary axis and cymose lateral branches, strawberry thyrse is distinct from inflorescences of other model species such as pea with compound racemes or petunia and tomato with monochasial cymes. General introduction of above-mentioned well-studied models and their inflorescences are included in the introduction while the differences and similarities of our findings in strawberry to these systems are handled in the discussion.**
- **Thyrse development has not been previously studied at the meristematic and molecular levels. We found that the primary and higher-order meristems have drastically different geometries but comparable size, and used these observations to provide mechanistic explanations of two key aspects of the strawberry inflorescence architecture: the production of single primordia by the round primary meristem vs. pairs of primordia by crescent-shaped higher-order meristems, and the erratic course of the main axis. The crescent shape of secondary and higher order IMs allows patterning of bracts and branches at the opposite poles of the meristem, resulting in typical dichasial cymose branching. Similar shape of the lateral meristems has not been observed in the previously studied systems, where the shape of the primary and secondary meristems is the same (e.g. petunia, pea). These issues are now better emphasized in the discussion.**
- **Compared to model systems studied until now, our data indicates a conserved role for FvFT1 in promoting FM identity, while we see differences but also similarities in FvTFL1 function. We have revised the discussion to highlight the observations that make strawberry distinct from the models with simple racemes (Arabidopsis), compound racemes (pea) and cymes (tomato). In contrast to other models, TFL1 expression is highest in the vegetative SAM where it acts as a strong repressor of flowering (Koskela et al. 2012), and downregulation of FvTFL1 is needed for the transition of the SAM into an IM. This contrasts with Arabidopsis and pea, where IM identity is associated with upregulation of TFL1 (in tomato TFL1/SP does not contribute to inflorescence development). We also observed further downregulation of TFL1 during the transition of IM to FM. This pattern in strawberry is similar to Arabidopsis: branching of the monopodial axis in both species can occur as long as there is some TFL1 activity left. FvTFL1 also increases the number branching iterations in the lateral branches but not their dichasial patterning. The specific features in strawberry are now emphasized in the abstract and in the discussion.**
- **While the antagonism between FT1 and TFL1 is not a new finding per se, it regulates the complexity of the woodland strawberry inflorescence in previously not investigated context of the thyrse template and involves unexpectedly different response of the primary meristem and higher-order meristems. To explain these features we proposed a model that is distinct from that postulated for Arabidopsis (transient changes of meristem states), and instead postulates (permanently) different identities for the primary and higher-order meristems. The primary meristem produces a variable number of lateral primordia; TFL1 and FT1 regulate how many of them will be produced before the transition to the flower state will take place. In contrast, higher-order meristems always produce a single pair of lateral primordia (as indicated by the bracts) and then deterministically acquire flowering identity; the developmental decision regulated by TFL1 and FT1 is whether these lateral primordia will develop into next-order branches or not. These novel aspects have been addressed in the discussion.**
- **The novelty of our work also includes methodological advances related to the modeling: First, Petri nets**

provide a more precise characterization of the development of inflorescences than the previously introduced flowcharts, because the nets offer a clear distinction between two processes: (i) a system's component choice to pursue one of several possible trajectories (e.g., the decision of a meristem to remain in the IM state or switch to the FM state), vs. (ii) the addition of a new component to the system, which will pursue its own trajectory concurrently with the parent (e.g., the production of a new primordium by an IM). Second, we illustrated the variability of inflorescence architectures using a realistic computational model, as opposed to diagrammatic models used for this purpose previously. The realistic representation facilitates comparisons with the actual plants and thus contributes to the validation of the model.

Reviewer #1 (Comments for the Author):

The ms provides a very good description of Fragaria inflorescence development, useful for researchers in the field and to understand developmental processes in this species.
The study on the phenotypic effects of altering the function or the activity of FvTFL1 and/or FvFT are well performed, are sound and well documented. They are convincing to show the antagonistic effect of FvTFL1 and FvFT in inflorescence complexity, supporting many previous studies in other species, but do not add, in my opinion, novel insights on the mechanisms of action, but are confirmatory, of course with the bonus of working with a different model system with a different type of architecture, therefore increasing somehow what we know these genes can do.

OUR RESPONSE: At this stage, and based on the data we have, we cannot give a detailed molecular-level explanation for the observed phenotypes, but our data and model do provide several insights regarding the mechanisms (see comments above). One of the key findings is that the primary and higher-order meristems respond differently to the TFL1 / FT1 dosage while always producing the thyrse architecture. This observation also suggests that there is an additional (upstream) factor that confers different identities on these meristems and constrains the observed architectures to thyrses. Our work thus rises an interesting research question.

The major novelty resides in the use of the data to feed a mathematical model that explains the development of the Fragaria inflorescence mainly on the parameter of vegetativeness, which here would be controlled by the opposite activities of FvTFL1 and FvFT. The model can be adjusted nicely to reproduce the observed variations in the inflorescence of WT, mutants and OX, something remarkable in this particularly elaborated architecture. However, while very nice and tackling a more complex situation, it again feels as a new example of a known mechanism (of course still valuable). I do not have any other comment, as, I said, I think that the experiments are well performed

OUR RESPONSE: There are actually novel aspects in the model as detailed above.

Reviewer #2:

This manuscript investigates the inflorescence architecture of woodland strawberry, Fragaria vesca, and shows that it is a thyrse, with racemose and cymose components. Using scanning electron microscopy to monitor the developmental progression of meristem transitions and maturation, the authors show that the strawberry thyrse results from differences in the size and shape of the primary and lateral inflorescence meristems. Importantly, the authors frame this architecture specification in the context of differing contributions by key regulators, FLOWERING LOCUS T and TERMINAL FLOWER 1. In addition, the authors present a computational model to support the opposing actions of FT and TFL1 as contributing to the phenotypic diversity observed across strawberry accessions.

Importance of findings

The authors unravel a complex inflorescence architecture in a herbaceous perennial and present the genetic basis for this complex pattern. Furthermore, they leverage computer modeling to support that timely changes in FT and TFL1 contribute to architectural diversity.

Quality of experiments

The experiments are excellent and complete. The developmental progression of meristems using scanning electron microscopy is very careful and thorough. The authors make use of mutant lines, many of which were previously characterized by this research team, and natural germplasm diversity. Relevant statistical analyses are shown.

Points in favor

This is a beautiful body of work, detailing an inflorescence architecture that I was not familiar with before reading this manuscript.

Points detracting

The authors' focus is a bit limited, and this is a complex story. The architectures of increasing numbers of crops are shown to result from contributions of FT and TFL1 and their homologs, and the significance of their impacts in strawberry may be better appreciated by a larger readership when put in this context.

OUR RESPONSE: We have brought up this issue at the end of the discussion to state how our results could be applied in the breeding of cultivated strawberry to optimize berry yield.

Line 184: so does tomato but this excellent work is not referenced (Tal et al, 2017).

OUR RESPONSE: We are aware of this indeed excellent work. However, to avoid confusion, we have not included this reference in line 184 (line 188 in revised version), as we are specifically focusing on results pertinent to strawberry there.

What is the difference between Reine des vallees vs Hawaii-4 in Fig. 3D vs E?

OUR RESPONSE: Both Reine des Vallees and Hawaii-4 have the same 2 bp mutation in the *TFL1* gene. Despite their different genetic backgrounds, they show the same inflorescence phenotype. Nevertheless, we agree that the original figure 3D could create confusion, and thus we have now replaced it by the Hawaii-4 phenotype (as in Fig. 3E).

TPC2022-RA-01077R1 2nd Editorial decision – *acceptance pending* **Feb. 26, 2023**

We are pleased to inform you that your paper entitled "Diversity of the woodland strawberry inflorescences results from heterochrony antagonistically regulated by FvTFL1 and FvFT1" has been accepted for publication in The Plant Cell, pending a final editorial review by a science editor.

Final acceptance from Science Editor **Mar. 14, 2023**
